# Endoscopic retrieval of fragmented pancreatic stent in pediatric pancreas divisum using pancreatoscopy

**DOI:** 10.1055/a-2686-3196

**Published:** 2025-09-18

**Authors:** Joao Guilherme Guerra, Carlos Alberto Ikoiti Takenaka, Isadora Amaral Savino Tenório Lisboa, José Celso Ardengh

**Affiliations:** 1 125097 Endoscopy Unit, Hospital São Camilo Santana, São Paulo, Brazil; 2 Endoscopy Unit, Hospital Moriah, São Paulo, Brazil

Fragmentation and migration of pancreatic stents pose significant challenges, especially in patients with complex anatomy such as pancreas divisum. The retention of foreign bodies within the pancreatic duct potentially leads to a diversity of complications like acute pancreatitis, fistula, and bloodstream infection 1 2 , and multiple methods of management have been described. We present a rare case of a 16-year-old female patient with recurrent acute pancreatitis due to a retained fragmented pancreatic stent ( Fig. 1 ), resulting from a previous removal attempt for complete pancreas divisum treatment.

**Fig. 1:**
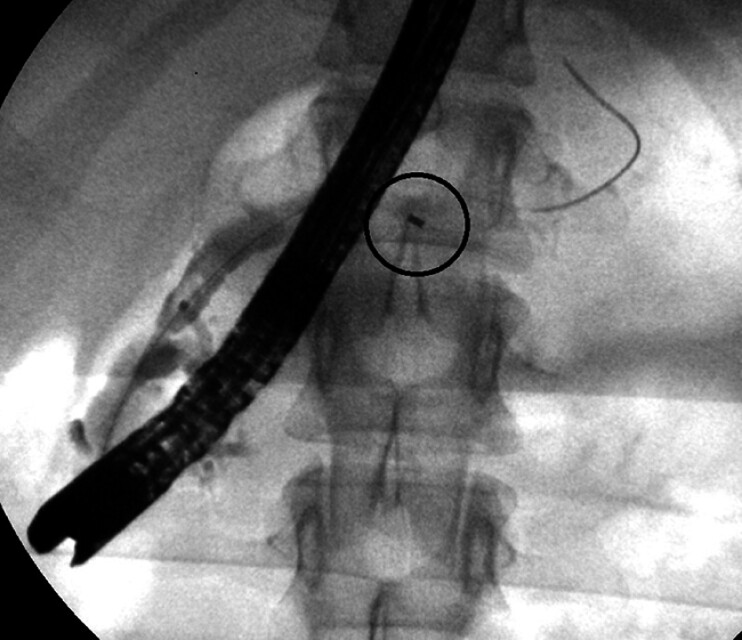
Retained stent fragment within the main pancreatic duct.

The patient, a 16-year-old female, experienced repeated episodes of acute pancreatitis after the fragmentation of a pancreatic stent during an ERCP 6 months prior. Endoscopic retrieval of the fragment was successfully performed using pancreatoscopy ( Video 1 , Fig. 2 ) with a cholangioscopy system and a dedicated foreign body retrieval snare ( Fig. 3 , Fig. 4 , Fig. 5 ). The procedure occurred without complications, and the patient recovered well, with no further pancreatitis episodes.

The video shows the retrieval of a plastic stent fragment retained after an ERCP to treat pancreatitis caused by pancreas divisum in a 16-year-old female. The fragment presence caused recurrent acute pancreatitis and was successfully retrieved by pancreatoscopy with no complications.Video 1

**Fig. 2:**
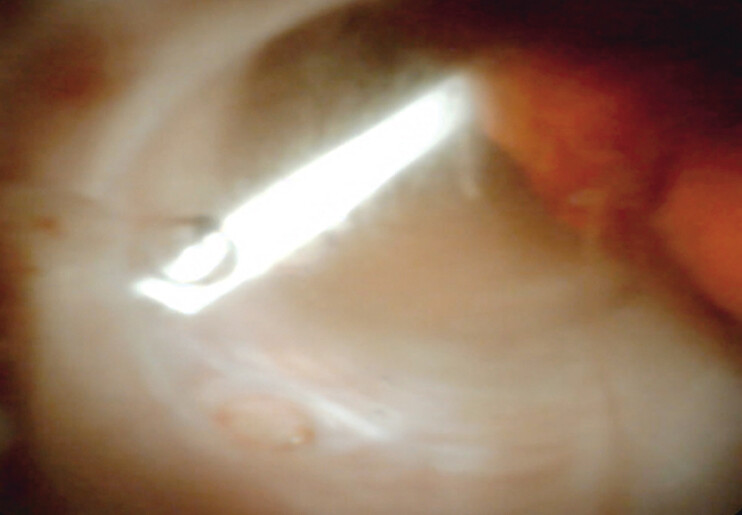
Pancreatoscopic view of the fragmented stent.

**Fig. 3:**
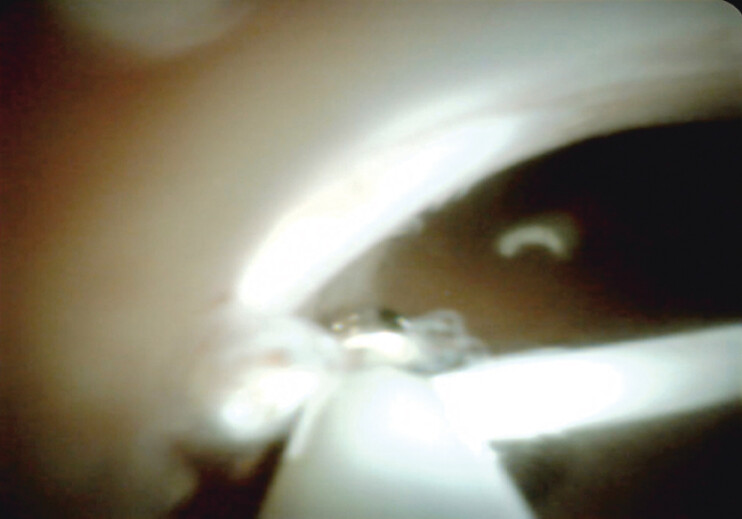
Fragmented stent grasped by retrieval snare.

**Fig. 4:**
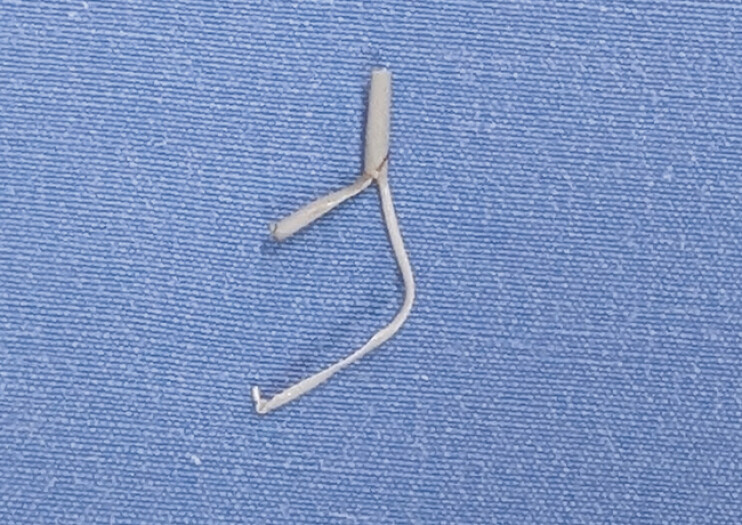
Retrieved stent fragment.

**Fig. 5:**
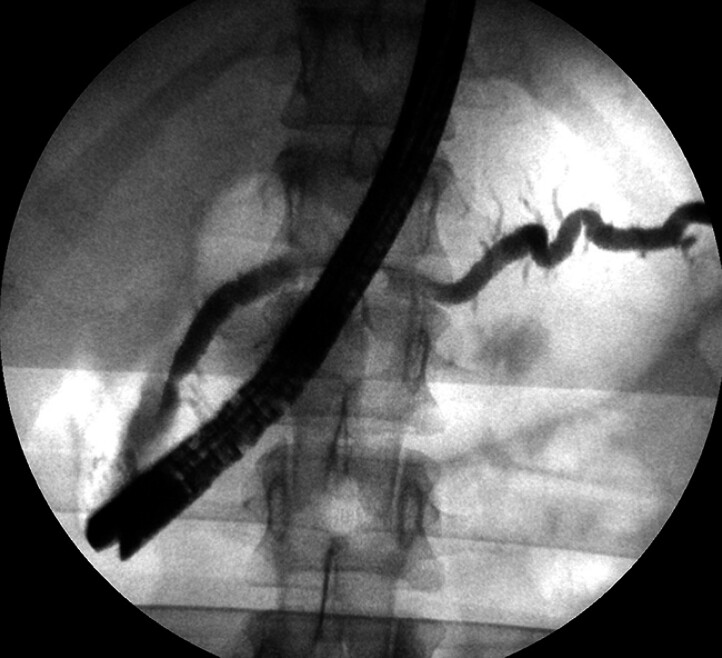
Final pancreatography with no residual stent.

This case highlights the efficacy of the cholangioscopy system-assisted pancreatoscopy in managing complex foreign bodies in the pancreatic duct, even in challenging pediatric anatomies. While stent fragmentation is documented 1 3 4 , its occurrence in a pediatric patient with pancreas divisum, requiring advanced endoscopic retrieval, is exceptionally rare 2 . Our successful outcome reinforces the growing capability of advanced endoscopic techniques for intricate clinical scenarios, underscoring the value of direct visualization for precise fragment removal 5 .

Endoscopy_UCTN_Code_TTT_1AR_2AI

## Notes

Endoscopy E-Videos https://eref.thieme.de/e-videos
*E-Videos* is an open access online section of the journal *Endoscopy* , reporting on interesting cases and new techniques in gastroenterological endoscopy. All papers include a high-quality video and are published with a Creative Commons CC-BY license. Endoscopy E-Videos qualify for HINARI discounts and waivers and eligibility is automatically checked during the submission process. We grant 100% waivers to articles whose corresponding authors are based in Group A countries and 50% waivers to those who are based in Group B countries as classified by Research4Life (see: https://www.research4life.org/access/eligibility/ ). This section has its own submission website at https://mc.manuscriptcentral.com/e-videos .
